# External and internal focus of attention differentially modulate corticospinal excitability in anticipatory postural adjustments

**DOI:** 10.1038/s41598-022-26987-1

**Published:** 2022-12-26

**Authors:** Amiri Matsumoto, Hajime Ueda, Akari Ogawa, Chihiro Oshima, Keisuke Irie, Nan Liang

**Affiliations:** Cognitive Motor Neuroscience, Human Health Sciences, Graduate School of Medicine, Kyoto University, 53 Shogoin-Kawahara-Cho, Sakyo-Ku, Kyoto, 606-8507 Japan

**Keywords:** Neurophysiology, Attention, Motor cortex

## Abstract

Whether attentional focus modulates the corticospinal excitability of the lower limb muscles in anticipatory postural adjustments (APAs) when performing a ballistic movement of the upper limb remains unclear. The present study used transcranial magnetic stimulation (TMS) to examine the corticospinal excitability of the lower limb muscles along with the kinematic profiles during dart throwing with different attentional foci, external focus (EF) and internal focus (IF). In 13 healthy participants, TMS was applied immediately before electromyographic onset of the tibialis anterior (TA) muscle, and the motor evoked potential (MEP) was recorded in the TA and soleus (SOL) muscles. The performance accuracy was significantly higher in the EF condition than in the IF condition. In both EF and IF conditions, MEP amplitude in the TA muscle, but not the SOL muscle, was significantly higher immediately before TA muscle onset (− 100, − 50, and 0 ms) compared to the control. In particular, the MEP increment in the TA muscle before TA muscle onset (− 50 and 0 ms) was significantly larger in the EF condition than in the IF condition. Our findings provide the first evidence for the modulation of corticospinal excitability in APA by changing attentional focus.

## Introduction

Attentional focus, which can be divided into external focus (EF) and internal focus (IF), affects motor performance^1^. In an EF strategy, performers concentrate on the movement outcome in the environment, whereas in an IF strategy, performers concentrate on their own body movements. Accumulating evidence indicates that compared with the IF strategy, the EF strategy enhances motor performance in force production^2,3^, balance^4,5^, jumping^6,7^, and accuracy^8,9^ in healthy populations and in reaching movements with the hemiparetic arm after stroke^10,11^.

Regarding the underlying mechanisms, neuroimaging studies have shown that the primary somatosensory cortex, premotor cortex, supplementary motor area, insular cortex, and intraparietal lobule may be activated during motor task with attentional focus^12–14^. Using a non-invasive method of transcranial magnetic stimulation (TMS)^15^, better motor performance in an EF is accompanied by enhanced levels of cortical inhibition and a more efficient neural strategy^16,17^. The short-interval intracortical inhibition in the primary motor cortex (M1) of the agonist muscle was greater with EF than with IF^16^, and the levels of surround inhibition in the M1 of the adjacent muscle were higher when adopting EF compared to IF during force control^17^. These results suggest that attentional focus modulates the excitability of the central nervous system (CNS), which corresponds to the agonist and adjacent muscles. However, it remains unclear whether attentional focus modulates the corticospinal excitability of the muscles not directly involved in the motor task, such as the postural muscles in the trunk or lower limb.

Perturbations from voluntary movements of the upper limb cause a shift in the centre of mass (COM) and impair postural equilibrium^18^. To minimise postural displacement from an expected perturbation in advance, the postural muscles in the trunk or lower limb are activated prior to those in the upper limb^19–21^. These postural reactions, known as anticipatory postural adjustments (APAs), are hypothesised to operate to maintain balance and prevent falls^22,23^. Such an unconscious process of APA is considered to be pre-programmed by the CNS^24,25^, and the M1 contributes to the control of APA in the trunk or lower limb when performing a rapid upper limb movement^21,26–28^. We recently investigated the modulation of corticospinal excitability in lower limb muscles (tibialis anterior [TA] and soleus [SOL] muscles) during ballistic and goal-directed upper limb movements. The TA muscle plays a role in APA in this scenario, namely, the preceding electromyographic (EMG) activity of the TA muscle in association with the increased corticospinal excitability in a muscle-specific manner^29^. The APA time was longer than that reported in the literature, which used a simple reaction task and single-joint movement, suggesting that the APA might be modulated depending on the difficulty of the motor task. Previous studies have reported that complex movements, rather than simple movements, led to increased excitability in motor-related areas involving the M1, sensorimotor cortex, supplementary motor area, premotor cortex, and somatosensory cortex^30–32^. Therefore, complex and goal-directed movements would involve more cognitive processes in association with motor commands than simple movements. However, it remains unknown whether the feedforward motor command related to APA is influenced by distinct cognitive processes involving generalised or directed attention in the motor task. Considering that the benefits of the EF strategy are not restricted to simple and single-joint movements and dynamic whole-body movements, of which the resulting motor performance involves contributions from postural muscles along with agonist and adjacent muscles, it seems plausible that postural muscle activity and corticospinal excitability can be modulated by different attentional foci.

The first aim of the present study was to investigate the attentional focus-dependent difference in motor performance in dart throwing using kinematic profiles and EMG activities. The second and the most important aim of the present study was to investigate the effects of the attentional focus on the corticospinal excitability of the postural muscles in the lower limb when performing dart throwing using TMS techniques. We hypothesised that the corticospinal excitability of the postural muscle contributing to APA is much enhanced with EF strategy than IF strategy, accompanied by differences in muscular activity, joint movement, and displacement of the centre of pressure (COP) if any.

## Results

### Motor performance and EMG activity

**Figure 1:**
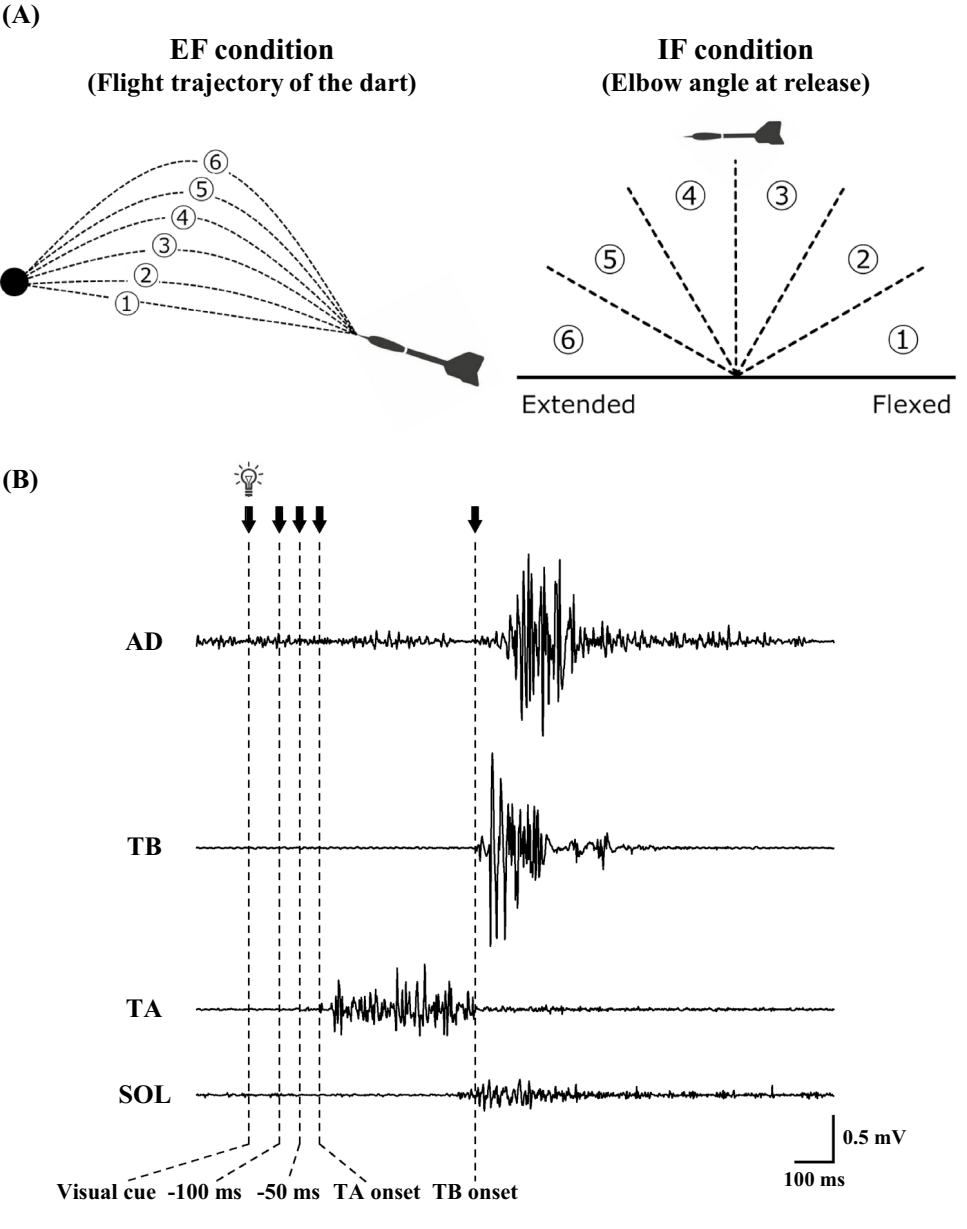
(**A**) Pictures provided in the experiments to ensure that the participants can keep their focus on the instructed target (EF condition, the flight trajectory of the dart [left]; IF condition, the elbow angle of the dominant hand at release [right]). (**B**) Original tracings demonstrating EMG activity of AD, TB, TA, and SOL muscles during dart throwing. Downward arrows indicate the timing of transcranial magnetic stimulation. *EF* external focus, *IF* internal focus, *EMG* electromyography, *AD* anterior deltoid muscle, *TB* triceps brachii muscle, *TA* tibialis anterior muscle, *SOL* soleus muscle.

**Figure 2:**
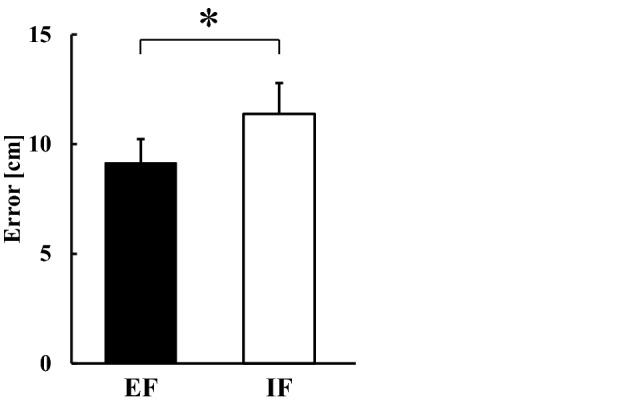
The performance error from the thrown dart to the bullseye calculated by the linear distance (N = 15). *EF* external focus, *IF* internal focus. *P < 0.05.

Manipulation check which evaluated how extent the participants concentrated on the instructed target showed no significant difference between the EF and IF conditions (EF, 7.9 [6–10]; IF, 7.6 [5–9]). The subjective ratings of performance were 2.6 (range 1–5) for ‘flight trajectory of the dart’ in the EF condition, and 3.9 (range 2–6) for ‘elbow angle at release’ in the IF condition (Fig. 1A). These results confirmed that the participants were able to concentrate on the instructed target well in both EF and IF conditions. Figure 2 shows the performance error calculated as the distance from the thrown dart. As previously reported^8,9^, the error was significantly smaller in the EF condition than in the IF condition (*P* < 0.05), indicating that the resulting performance accuracy is significantly higher in the EF condition than in the IF condition.

**Figure 3:**
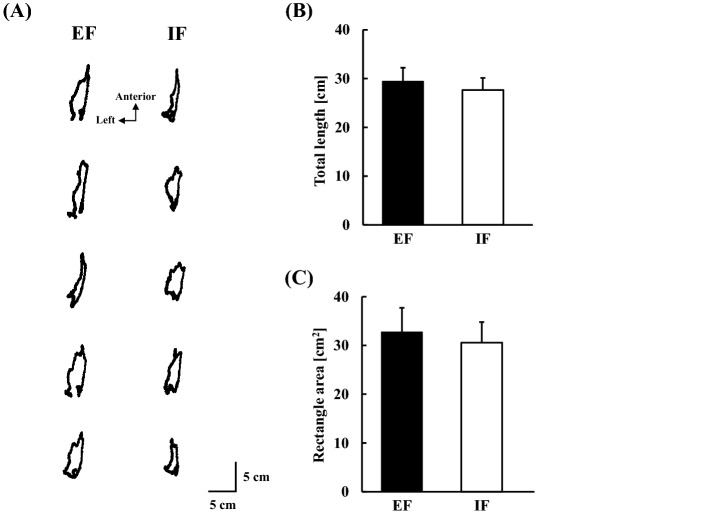
Displacement of COP within 3 s from the visual cue (N = 15). (**A**) Representative COP recordings (five consecutive trials) with EF and IF conditions, respectively. (**B**) The total length of COP is calculated with EF and IF conditions. (**C**) The rectangular area of COP is calculated with EF and IF conditions. *COP* centre of pressure, *EF* external focus, *IF* internal focus.

**Figure 4:**
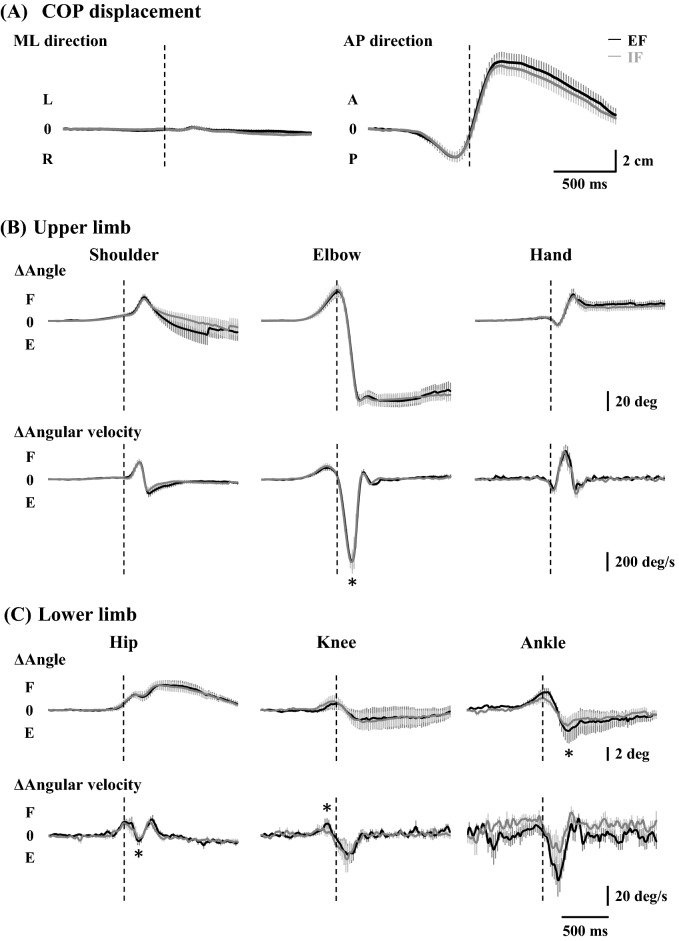
Kinematic parameters in the time course. (**A**) Displacement of COP (N = 15). (**B**,**C**) Joint movements in the upper and lower limbs, respectively (N = 9). All data were aligned to the EMG onset of the TB muscle (vertical dotted lines). *EF* external focus, *IF* internal focus, *EMG* electromyography, *TB* triceps brachii muscle, *COP* centre of pressure, *A* anterior, *L* left, *P* posterior, *R* right, *F* flexion, *E* extension. *P < 0.05 significant difference between EF and IF conditions.

Representative COP recordings under both EF and IF conditions are shown in Fig. 3A, and the average changes in the displacement of COP over time are shown in Fig. 4A. There were no statistical differences in the total length and rectangular area of COP between the EF and IF conditions (Fig. 3B,C). The onsets of posterior and anterior movements were similar between conditions (Table 1), and there was no significant difference in the backward or forward, left or right COP peak between the EF and IF conditions, respectively (backward: EF, 4.0 ± 0.5 cm, IF, 3.8 ± 0.5 cm; forward: EF, 7.0 ± 0.8 cm, IF, 6.5 ± 0.6 cm; left: EF, 0.8 ± 0.1 cm, IF, 0.8 ± 0.1 cm; right: EF, 0.9 ± 0.1 cm, IF, 0.9 ± 0.1 cm).

**Table 1:** The onsets of kinematic parameters and EMG activities aligned to the TB muscle onset.

	Time (ms)	
	EF	IF
**Displacement of COP**		
COP-posterior	− 306.1 ± 34.9	− 296.9 ± 31.8
COP-anterior	− 74.8 ± 26.3	− 81.2 ± 25.4
**Elbow joint movements**		
Elbow flexion	− 260.9 ± 35.5	− 271.1 ± 34.2
Elbow extension	20.4 ± 6.5	14.2 ± 5.1
**EMG**		
AD	52.4 ± 9.4	62.7 ± 11.8
TA	− 332.7 ± 33.3	− 283.6 ± 26.2
SOL	− 13.6 ± 16.7	− 16.2 ± 19.9

Values are means ± SE. *EMG* electromyography, *TB* triceps brachii muscle, *EF* external focus, *IF* internal focus, *COP* centre of pressure, *AD* anterior deltoid muscle, *TA* tibialis anterior muscle, *SOL* soleus muscle.

Table 2 summarises the maximum changes in the angle and angular velocity of all joint movements, and the average changes over time are shown in Fig. 4B,C. In the upper limb for throwing, the change in angular velocity of elbow extension was significantly larger in the IF condition than in the EF condition (*P* < 0.05, Table 2 and Fig. 4B). There were no statistical differences in the onset of elbow flexion and extension in the time course between conditions (Table 1). In the lower limb for postural control, the changes in the angular velocity of hip extension and knee flexion and the change in the angle of plantar flexion were significantly larger in the EF condition than in the IF condition (*P* < 0.05, respectively; Table 2 and Fig. 4C).

**Table 2:** Kinematic information during dart throwing.

Joint	Movement	ΔAngle (deg)		ΔAngular velocity (deg/s)	
		EF	IF	EF	IF
Shoulder	Flexion	27.4 ± 3.5	26.0 ± 2.6	295.1 ± 33.3	307.7 ± 37.6
	Extension	1.4 ± 0.7	0.8 ± 0.3	198.4 ± 36.7	177.8 ± 32.5
Elbow	Flexion	31.3 ± 5.8	33.0 ± 6.0	153.9 ± 29.4	162.3 ± 30.3
	Extension	89.5 ± 5.1	90.1 ± 6.0	1141.1 ± 76.8 *	1192.1 ± 81.5
Hand	Palmar flexion	32.9 ± 6.0	33.3 ± 4.6	539.8 ± 72.2	582.1 ± 57.0
	Dorsal flexion	10.5 ± 1.9	11.4 ± 2.4	358.3 ± 65.8	440.1 ± 43.1
Hip	Flexion	4.3 ± 0.7	4.0 ± 0.8	36.5 ± 4.2	34.8 ± 4.4
	Extension	0.7 ± 0.2	0.6 ± 0.3	25.8 ± 2.7 *	21.7 ± 2.5
Knee	Flexion	3.4 ± 0.9	2.6 ± 0.8	41.4 ± 7.2 *	29.9 ± 4.1
	Extension	3.6 ± 1.3	3.1 ± 1.1	49.3 ± 11.3	49.4 ± 11.4
Ankle	Dorsal flexion	3.8 ± 0.7	3.0 ± 0.6	53.1 ± 7.8	56.2 ± 6.7
	Plantar flexion	5.1 ± 1.7 *	3.3 ± 1.0	92.6 ± 22.6	67.5 ± 13.5

Values are means ± SE. *Indicates the significant difference (*P* < 0.05) between EF and IF conditions. *EF* external focus, *IF* internal focus.

Representative EMG recordings are shown in Fig. 1B. The EMG onset timings of the anterior deltoid (AD), TA, and SOL muscles aligned with the EMG onset of the triceps brachii (TB) muscle are summarises in Table 1. Two-way analysis of variance (ANOVA) with repeated measures showed a significant effect of the ‘muscle’ (*F*_(3,42)_ = 174.8, *P* < 0.0001, η^2^ = 0.81), but no effect of the ‘condition’ (*F*_(1,14)_ = 2.8, *P* = 0.10, η^2^ = 0.002), with no interaction between the factors (*F*_(3,42)_ = 2.0, *P* = 0.13, η^2^ = 0.004). In both EF and IF conditions, the EMG activity of the TA muscle significantly preceded the onset of the TB muscle (*P* < 0.0001), while that of the SOL muscle started slightly earlier (but not significantly) than that of the TB muscle (*P* = 0.43). On the other hand, the onset of the AD muscle was significantly delayed (*P* < 0.001). Regarding the integrated EMG (iEMG) activity in each muscle, two-way ANOVA with repeated measures showed no effect of the ‘condition’ (*F*_(1,14)_ = 3.0, *P* = 0.09, η^2^ = 0.002; TB muscle, EF: 46 ± 4% of maximum voluntary contraction [MVC], IF: 52 ± 6%MVC; TA muscle, EF: 11 ± 2%MVC, IF: 9 ± 1%MVC; SOL muscle, EF: 27 ± 6%MVC, IF: 25 ± 5%MVC), but the AD muscle (EF, 55 ± 8%MVC; IF, 59 ± 9%MVC) showed a trend of less activity in the EF condition than in the IF condition (post hoc, *P* = 0.06).

### Corticospinal excitability during APA with external and internal focus conditions

**Figure 5:**
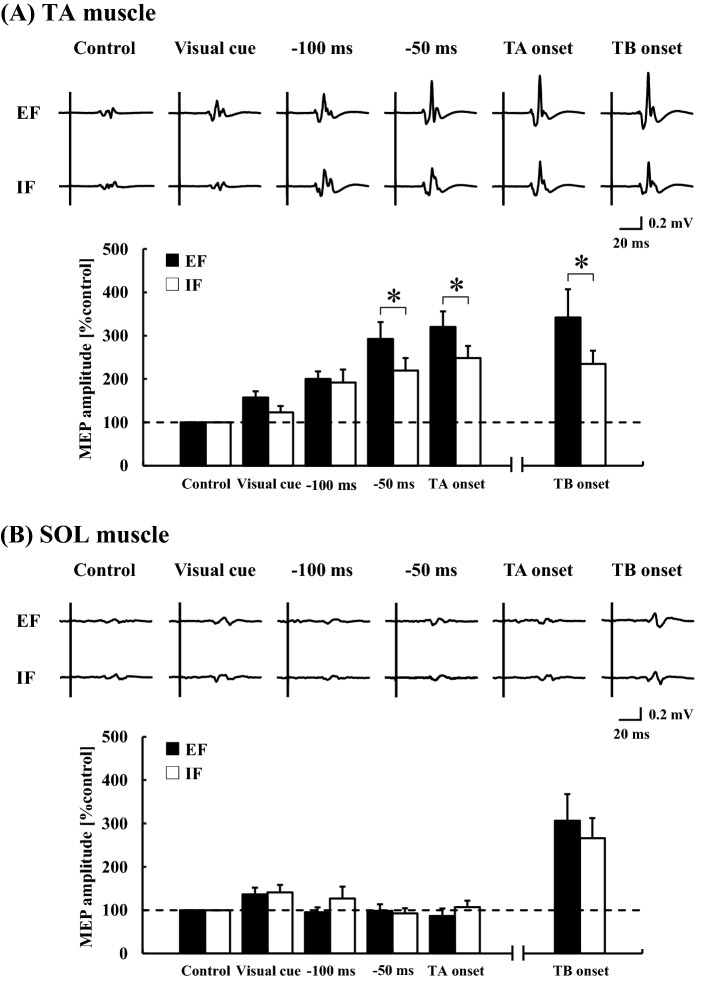
Representative recordings of MEP (averaged five trials, respectively) and the average changes at the time points in the TA muscle (**A**, N = 13) and the SOL muscle (**B**, N = 12) with EF and IF conditions. Vertical solid lines in the raw traces indicate the stimulus pulse of transcranial magnetic stimulation. *MEP* motor evoked potential, *TA* tibialis anterior muscle, *SOL* soleus muscle, *TB* triceps brachii muscle, *EF* external focus, *IF* internal focus. *P < 0.05 significant difference between EF and IF conditions.

Representative motor evoked potential (MEP) recordings and group mean data in the TA and SOL muscles are shown in Fig. 5A,B, respectively. In the TA muscle, two-way ANOVA with repeated measures showed a significant effect of the ‘condition’ (*F*_(1, 12)_ = 18.4, *P* < 0.0001, η^2^ = 0.03) and ‘time point’ (*F*_(4, 48)_ = 30.7, *P* < 0.0001, η^2^ = 0.38), and significant interaction between the factors (*F*_(4, 48)_ = 3.1, *P* = 0.02, η^2^ = 0.02). In both EF and IF conditions, MEP significantly increased at − 100 ms (*P* < 0.005, respectively), − 50 ms (*P* < 0.001, respectively), and TA onset (*P* < 0.0001, respectively) compared to that at control (EF, 0.27 ± 0.06 mV; IF, 0.30 ± 0.07 mV), whereas there was no significant increase in the MEP at visual cue (EF, *P* = 0.17; IF, *P* = 0.80). Importantly, MEP was significantly greater in the EF condition than in the IF condition at − 50 ms and TA onset (*P* < 0.01, respectively). MEP at TB onset also significantly increased in both conditions (*P* < 0.01, respectively; background EMG [B.EMG] activity, EF: 3.5 ± 0.8%MVC, IF: 3.0 ± 0.9%MVC) and was significantly larger in the EF condition than in the IF condition (*P* = 0.02).

MEP was also successfully recorded in the SOL muscle in 12 of the 13 participants (Fig. 5B). Two-way ANOVA with repeated measures showed a significant effect of the ‘time point’ (*F*_(4, 44)_ = 5.4, *P* = 0.003, η^2^ = 0.09), but no effect of the ‘condition’ (*F*_(1, 11)_ = 2.1, *P* = 0.15, η^2^ = 0.009) and interaction between the factors (*F*_(4, 44)_ = 0.99, *P* = 0.42, η^2^ = 0.02). There were no significant changes in the MEP at − 100 ms, − 50 ms, and TA onset compared to that at control (EF, 0.24 ± 0.05 mV; IF, 0.23 ± 0.05 mV). MEP at TB onset was significantly higher compared to that in the control in both conditions (*P* < 0.01, respectively), but there was no significant difference between conditions (*P* = 0.32).

## Discussion

In the present study, we investigated the effects of attentional focus on corticospinal excitability of the lower limb muscles in the APA phase using a complex and goal-directed motor task of dart throwing. The major finding was that the corticospinal excitability of the TA muscle significantly increased prior to muscle contraction in both EF and IF conditions, and that the increment was significantly greater in the EF condition than in the IF condition. To the best of our knowledge, the present study provides the first evidence that attentional focus may play an important role in the modulation of corticospinal excitability in APA.

### Motor performance during APA with different attentional foci

APA with lower limb, upper limb, and trunk muscles operates for human voluntary movements, and most previous studies used simple and/or single-joint movements to investigate APA^18–21^. We adopted a more complex and goal-directed movement of dart throwing to ensure that participants easily changed their attentional focus with the movement. The resulting performance accuracy was significantly greater in the EF condition than in the IF condition, in line with previous studies^1^. Subsequently, it is of interest to determine whether joint movements of the upper and lower limbs and COP during APA differ depending on the focus of attention applied.

The COP shifted in a posterior direction at a significantly early stage accompanied by a burst of TA muscle after the visual cue (Table 1), and it might be triggered in advance by slight elbow flexion before full elbow extension, as previously reported^29^. The angular velocity of elbow extension was greater in the IF condition than in the EF condition, and this is reasonable in terms of attentional focus. Because participants concentrated on the elbow joint in the IF condition, they might throw a dart by mainly extending the elbow joint, resulting in acceleration of the angular velocity of the elbow extension with less performance accuracy (Fig. 2), according to the theory of speed-accuracy trade-off^33^. An increase in the velocity of the intended movement might affect APA in duration and/or magnitude, as suggested by a previous study^34^. Conversely, the changes in the angular velocity of the hip and knee joints and the change in the angle of the ankle joint were slightly greater in the EF condition than in the IF condition. These results suggest that participants throw the dart using whole-body movement with emphasis on the lower limb when they concentrate on the flight trajectory of the dart in the EF condition. Interestingly, despite the differences in the kinematic results of the upper and lower limbs, the COP showed no significant differences between the EF and IF conditions. Because the displacement of COP reflects whole-body movement, including the upper limb, trunk, and lower limb, it is likely that the perturbations from the throwing movement and postural control were counteracted and showed no difference between the EF and IF conditions.

### EMG activities during APA with different attentional foci

Previous studies have reported that the agonist and/or antagonist muscles showed less activity under EF than IF conditions in force production^35,36^ and dart throwing task^37^. In the present study, the AD muscle (synergist) showed a trend of less activity in the EF condition than in the IF condition, suggesting that EF might lead to efficient coordination of the agonist and/or synergist muscles and might result in better performance accuracy and less muscular activity of the AD muscle in dart throwing.

The upper limb movement of dart throwing including a slight elbow flexion followed by a full elbow extension would cause a slight posterior followed by an anterior shift of COM. Prior to these upper limb movements, COP would shift in a posterior and an anterior direction, respectively. As shown in the Fig. 4 and Table 1, the TA muscle activity and subsequent posterior COP shift started preceding the elbow flexion, which caused the anterior shift of COM. This preceding anterior-shifted COM might be responsible for counteracting or minimising the upcoming posterior shift of COM by the elbow flexion to maintain the postural stability. At this early stage, the TA muscle activity might be an anticipatory postural reaction to stabilize the body sway from the elbow flexion of dart throwing in advance. Thereafter, the deactivation of the TA muscle and/or activation of the SOL muscle caused the anterior-shift of COP and decelerated the anterior-shift of COM, namely a posterior-shift of COM. These sequential changes in the TA and SOL muscle activities as well as COP would operate for counteracting the upcoming, strong and anterior shift of COM by elbow extension. In particular, the SOL muscle onset was observed after the elbow flexion movement and showed no significant difference from the TB muscle onset, suggesting that the activity of this antigravity muscle was interpreted as a compensatory, but not an anticipatory, postural reaction during dart throwing.

In spite of these sequential and complex COP-COM dynamics during dart throwing, there was no significant difference in burst timing and amount of TA or SOL muscle between the EF and IF conditions. At a performance level, previous work utilising a Fitts’ task in the lower limb (i.e. a fast one-leg movement) showed that EF led to better motor performance, longer APA duration, and smaller APA magnitude than IF^38,39^, suggesting that the standing posture might be controlled more efficiently when adopting an EF strategy. The difference in motor task or interlimb/intralimb coordination might explain the difference between the previous and present results. In a previous study, the postural muscles were directly involved in the motor task, whereas a complex and goal-directed upper limb movement was used to explore the APA in the lower limb muscles, which were not directly involved in the motor task in the present study. There were no significant differences in the APA duration and magnitude between the EF and IF conditions, whereas the kinematic results showed greater changes in the angle and angular velocity of the lower limb in the EF condition, as mentioned above. One explanation for these observations is that the comparable magnitude of lower limb EMG activity with larger joint movements with an EF strategy might lead to more effective postural control than an IF strategy during dart throwing. In addition to the TA and SOL muscles, other lower limb muscles for extension of the hip joint and/or flexion of the knee joint might contribute to the significance between the EF and IF conditions. This is purely hypothetical, and further studies are required to address this issue.

### Corticospinal excitability in APA with different attentional foci

Consistent with our previous report^29^, the corticospinal excitability of the TA muscle, which contributes to APA in dart throwing, significantly increased before the EMG burst under both EF and IF conditions. The corticospinal excitability of the SOL muscle showed no change, suggesting that the corticospinal tract play a role in APA in a muscle-dependent manner. Importantly, MEP increments in the TA muscle prior to TA onset and at TB onset were significantly larger in the EF condition than in the IF condition (Fig. 5), indicating differential regulation of corticospinal excitability immediately prior to and during the APA phase. Considering that the angle and angular velocity changes in the lower limb were significantly greater in the EF condition than in the IF condition, the increased corticospinal excitability of the TA muscle in the EF condition would contribute to controlling the subsequent greater movement of the lower limb during throwing movement.

Previous studies using TMS reported no significant difference in the corticospinal excitability of the agonist muscle between the EF and IF conditions in finger^16,17,40^ and elbow^41^ movements. However, the short-interval intracortical inhibition in the M1 of the agonist muscle was greater in the EF condition than in the IF condition, and the levels of surround inhibition in the adjacent muscle were higher when adopting an EF compared to an IF during force control^16,17^. Moreover, adopting an IF strategy showed higher excitability of slow motor pathways, but not fast motor pathways, in comparison with the EF strategy^40^. Recently, an electroencephalogram (EEG) study further revealed that the IF condition increased the EEG coherence (10–12 Hz) of Alpha 2 between T3 (verbal-analytical region) and Fz (motor planning region) compared to that without any instruction regarding attentional focus, suggesting that an IF strategy might lead to higher real-time conscious motor processing and reduce the accuracy of motor performance^42^. These results highlighted a dissociation between corticospinal and cortical excitability corresponding to the agonist muscle and suggested that better motor performance in the EF condition was accompanied by enhanced levels of cortical inhibition and a more efficient neural strategy. Our results further revealed that the corticospinal excitability of the postural muscles engaged in APA increased, suggesting that attentional focus potentially modulates corticospinal excitability in the APA muscle. The larger increment of MEP in the EF condition compared with the IF condition may reflect a stronger neural drive (central motor command) originating in higher brain centres.

Multiple descending pathways link the cortex to the spinal cord, enabling the transmission of central motor commands for voluntary movement to spinal motoneurons. MEP to TMS mainly reflects the excitability of the corticospinal tract at cortical and spinal levels. Using single-pulse TMS without direct measurement of cortical or spinal excitability separately, it is difficult to identify the origin responsible for our results. Previous studies using combinations of single- and paired-pulse TMS, cervicomedullary stimulation, and H-reflex have shown that the M1 would be involved in APA control^21,26^. In addition, the MEP of the TA muscle in the present study were clearly facilitated in the absence of EMG activity prior to TA onset, and a distinct difference was observed between the EF and IF conditions. The amount of MEP enhancement prior to TA onset without EMG activity was comparable to that at TB onset, in which EMG activities were always involved, suggesting an excitability change at the supraspinal level rather than at the spinal level. Taking into consideration the previous and present results, it is most likely that the APA in the lower limb was pre-programmed by the CNS, and the differential enhancements of MEP depending on the cognitive state (EF and IF conditions) might be attributed to excitability changes at the supraspinal level, such as the M1. Other cortical regions involving the primary somatosensory cortex, premotor cortex, supplementary motor area, insular cortex, and intraparietal lobule are also candidates for contributing to the difference between the EF and IF conditions^12–14^. Interestingly, the supplementary motor area is also involved in APA^43,44^, and it may play a role in mediating the M1 or corticospinal excitability during APA with different attentional foci. Therefore, future neuroimaging studies are warranted.

Apart from the corticospinal tract or cerebral cortex, the reticulospinal tract is a candidate for the modulation of motor neuron excitability. In the literature, the primate reticulospinal tract is usually considered to control proximal and axial muscles and is involved mainly in gross movements, such as locomotion, reaching, and posture^45^. The reticular formation of the brain stem processing sensory input and guiding motor output is hypothesised to be responsible for APA. During voluntary movements, because the central motor command activates the spinal motor neuron and the neural circuits in the brain stem concomitantly, the increased M1 excitability by the voluntary drive of dart throwing may activate the spinal motoneuron pool of the TA muscle directly through the corticospinal tract and indirectly through the corticoreticular tract.

### Limitations

The present study has some limitations that should be acknowledged. First, no control condition (without instruction of attentional focus) was adopted in the present study. Our recent study^29^ has suggested that it was difficult to make a neutral focus condition, because the participants paid attention with the motor task either on the movement outcome and external environment (EF) or their own body and movement (IF) consciously or unconsciously. To elucidate whether the corticospinal excitability is modulated differentially by EF and IF, we adopted the EF and IF conditions in order to distinguish the attentional focus condition clearly in the present study. Second, the motor performance involving kinematic and EMG profiles during dart throwing was measured in a separate protocol without TMS to precisely determine the behavioural difference between the EF and IF conditions. TMS using the double cone coil would affect the following motor performance because the stimulation would spread in the M1 and induce muscle activation in other trunk and/or upper limb muscles apart from the TA and SOL muscles. Third, it was difficult to precisely clarify the temporal changes in corticospinal excitability in the SOL muscle because TMS was applied over the motor hotspot of the TA muscle and stimulus timings were determined by the TA muscle onset. According to our results, the corticospinal excitability of the SOL muscle remained unchanged immediately before TA muscle onset, whereas that of the TA muscle increased. Finally, because the cortical and spinal excitability were not measured separately, it was difficult to identify the origin responsible for our results. According to our results and previous studies^16,17,21,26^, it is likely that differential modulations of the corticospinal excitability between the EF and IF conditions in APA are originated from the excitability changes at the cortical level. Further studies are required to clarify this.

## Conclusions

The present study demonstrates for the first time that adopting an EF leads to better motor performance and to higher activity of the corticospinal excitability of the lower limb muscle in APA. Our findings suggest that attentional focus modulates the corticospinal excitability of the postural muscle, which is not directly involved in motor tasks.

## Methods

### Participants

Fifteen healthy volunteers (nine women; mean age, 23.9 ± 3.7 years) who did not meet the exclusion criteria for undergoing TMS^46^ and were not diagnosed with any known neurological or orthopaedic disorders participated in the present study. None of the participants were professional dart players. Fourteen participants were right-handed, as assessed using the Flinders Handedness survey questionnaire (9.6 ± 1.1 points)^47,48^, whereas the remaining one was ambidextrous (0 points), who always throws objects using the right hand. Motor performance, including displacement of COP and EMG activity, was assessed without TMS in all participants (protocol 1). In nine of the 15 participants, we additionally assessed joint movements using the three-dimensional motion analysis in protocol 1. Thirteen of the 15 participants were recruited in the TMS protocol (protocol 2). All participants provided written informed consent prior to the experiments. The experimental procedures and protocols were approved by the Ethics Committee of Kyoto University Graduate School and Faculty of Medicine and were performed according to the Declaration of Helsinki.

### Experimental procedures

The general experimental settings and equipment have been described previously^29^. Briefly, participants were asked to stand upright with their feet closed and face the dart board straight, with their right shoulder and elbow joints flexed approximately 90° (starting position), and then to throw a dart with their right hand in response to a visual cue (LED light).

Participants received EF or IF instruction before each block in the present study (see the “Experimental protocols”). In the EF condition, participants were instructed to ‘concentrate on the flight trajectory of the dart and throw the dart as forcefully as possible, aiming at the centre of the board (bullseye) after the visual cue’. In the IF condition, they were instructed to ‘concentrate on the elbow angle of the dominant hand and throw the dart as forcefully as possible, aiming at the bullseye after the visual cue’. Pictures (Fig. 1A, modified from Ref.^49^) were provided to the participants during the experiments to help them maintain their focus on the instructed strategy. In the EF condition, participants were instructed to rate the flight trajectory of the dart on a scale from 1 (straight line) to 6 (parabola). In contrast, in the IF condition, they were instructed to rate their elbow angle at release on a scale from 1 (fully flexed) to 6 (fully extended). The subjective rating of performance was reported after each throw in the EF or IF condition. As a manipulation check, participants were asked to rate how extent they concentrated on the instructed target on a scale from 0 (not at all) to 10 (perfectly) after each block.

### Motor performance

Kinematic performance was assessed using three-dimensional motion analysis (KinemaTracer system, Motion Recorder, KISSEI COMTEC Corporation Ltd., Japan). Four cameras were placed on the right side of each participant. The reflective markers were attached to the acromion, lateral epicondyle of the humerus, ulnar styloid, and fifth metacarpal head in the right upper limb, and to the greater trochanter, lateral epicondyle of the knee, lateral malleolus, and fifth metatarsal head in the right lower limb. Changes in the angle and angular velocity in the right upper and lower limb joints were recorded at a sampling rate of 50 Hz (3D Calculator, KISSEI COMTEC Corporation Ltd., Japan). A force plate (TF-6090, Tec Gihan Corporation Ltd., Japan) under the participant’s feet was used to record COP (sampling rate, 50 Hz) (Vital Recorder 2, KISSEI COMTEC Corporation Ltd., Japan).

### Surface EMG recordings

EMG was recorded from the right AD, long head of the TB, TA, and SOL muscles with a pair of silver-bar electrodes (Bagnoli-4 EMG System, Delsys, USA). Electrodes were placed over the belly of each muscle, and the reference electrode was attached to the right olecranon. The EMG signals were amplified and band-pass filtered at 20 Hz–2 kHz. Before the experiment, participants were asked to maximally perform the shoulder flexion, elbow extension, dorsal and plantar flexion for 2–3 s, and the MVC per second for each muscle was calculated for normalisation.

### Transcranial magnetic stimulation

A magnetic stimulator (Magstim 200^2^, The Magstim Company Ltd., Whitland, UK) with a double-cone coil (130 mm external diameter of wings) was used to deliver TMS. The coil was placed around the vertex, and the stimulus intensity by which TMS could induce an identifiable MEP in the right TA muscle was used when searching the motor hotspot. Once the motor hotspot of the TA muscle was determined, we fixed the coil position and the optimal site was marked with a pen on the swimming cap-covered scalp. A monophasic current in a posterior–anterior direction was applied to the left M1. The resting motor threshold (rMT) of the TA muscle was defined as the lowest stimulus intensity of TMS evoking an MEP of above 50 µV in amplitude in more than four of eight trials^50^. The stimulus intensity was set at 110–120% of rMT to induce an identifiable MEP in the TA muscle in resting state. In 12 of the 13 participants, MEP in the SOL muscle was robustly visible (> 50 μV) and was then also recorded and analysed.

### Experimental protocols

Before the main experiment, 5–10 trials of dart throwing were performed without instruction on attentional focus to familiarise the participants with the motor task. In protocol 1, without TMS, the participants were instructed to throw the dart with the EF or IF strategy after a visual cue of which the timing was randomised. Each block included five trials with EF or IF condition, and the participants performed two blocks (10 trials) for each condition (a total of 20 trials). The order of the EF and IF blocks was counterbalanced between the participants. The performance accuracy was assessed after each block (approximately 3–5 min). We also assessed joint movement, COP displacement, and EMG activity. EMG onsets of the TB and TA muscles were calculated and used to determine the TMS timings in protocol 2.

TMS was provided at several time points during dart throwing in protocol 2 (Fig. 1B). Five to 10 responses (MEPs) were recorded at each time point, and the order of the time points was randomised between the participants. The time points prior to the postural muscle activity (visual cue, − 100 ms, − 50 ms, and TA onset) and at the onset of agonist muscle activity (TB onset), which were determined by the averaged individual reaction time without TMS, were adopted in the TMS sessions according to the previous studies^21,26^. Except for the time point of TB onset, we confirmed that there was no EMG activity in the TA muscle prior to the TMS trigger. At these points, the number of the trials involved in the analysis was 38.5 ± 2.2 trials with EF condition (6.0 ± 0.7 trials for each time point) and 39.7 ± 2.6 trials with IF condition (6.0 ± 0.7 trials) after excluding the trials involving significant B.EMG activity in the TA muscle. MEPs recorded while the participants stood in a resting state was regarded as the control condition.

### Data analysis

In protocol 1, the performance accuracy was measured by the linear distances from the thrown darts to the bullseye. The angle and angular velocity of each upper or lower limb joint and the force plate signal (COP) were recorded and stored on a computer for offline analysis. These kinematic data were calculated by the changes from those in the starting position, within 3 s from the visual cue. A data acquisition software (LabChart, ADInstruments, Sydney, Australia) with a PowerLab analogue-to-digital converter (PowerLab 8/30, AD Instruments, Sydney, Australia) was used to record the EMG activities with a sampling rate of 4 kHz. The value of mean + 2SD within a 100-ms window before a visual cue for each participant was used as a cutoff value to determine the onset of the EMG activities. The average values of iEMG activities during the motor task were calculated and presented as a percentage of MVC (%MVC). All time-course data were realigned to the EMG onset of the TB muscle (defined as 0 ms).

In protocol 2, the peak-to-peak values of MEPs were measured and normalised as a percentage of those in the control condition. The results of MEP were grouped by each time point. The B.EMG activities (within a 100-ms window) prior to the TMS trigger were calculated, and the value of mean + 2SD in control condition for each participant was used as a cutoff value to determine the significant B.EMG activities. The trials involving significant B.EMG activities in the TA muscle were omitted from the analysis, except at the time point of TB onset.

### Statistical analyses

All data were analysed using JMP Pro 15 (SAS Institute Inc., Cary, North Carolina, USA). The normal distribution was tested by the Shapiro–Wilk test and the Mauchly test was used to test the sphericity. The Greenhouse–Geisser ε correction was used to evaluate the F-ratios for repeated measures. In protocol 1, the performance accuracy, the changes in the angle and angular velocity, the backward, forward, left, and right COP shifts, and the total length and rectangular area of COP were analysed using a paired *t*-test (EF and IF). The manipulation check questionnaire was analysed with the Wilcoxon signed-rank test (EF and IF). Two-way ANOVA with repeated measures was used to determine the difference in the EMG onset timing and iEMG activity (factors, condition and muscle), followed by a paired *t*-test with Holm’s sequential Bonferroni correction^51^. In protocol 2, MEP amplitudes in the TA and SOL muscles at control, visual cue, − 100 ms, − 50 ms, and TA onset were analysed using two-way ANOVA with repeated measures (factors, condition and time point), followed by Dunnett’s test. MEP amplitudes at TB onset and control were compared using a paired *t*-test. The effect size for ANOVA was calculated using eta squared (η^2^)^52^. The level of statistical significance was set at *P* < 0.05. Results are presented as the mean ± standard error for parametric data and presented as the mean (range) for nonparametric data.

## Data Availability

The datasets for the current study are available from the corresponding author on reasonable request.
